# Entropy and Turbulence Structure

**DOI:** 10.3390/e24010011

**Published:** 2021-12-22

**Authors:** T.-W. Lee, J. E. Park

**Affiliations:** Mechanical and Aerospace Engineering, SEMTE, Arizona State University, Tempe, AZ 85287, USA; jpark250@asu.edu

**Keywords:** turbulence, energy spectra, scaling, maximum entropy

## Abstract

Some new perspectives are offered on the spectral and spatial structure of turbulent flows, in the context of conservation principles and entropy. In recent works, we have shown that the turbulence energy spectra are derivable from the maximum entropy principle, with good agreement with experimental data across the entire wavenumber range. Dissipation can also be attributed to the Reynolds number effect in wall-bounded turbulent flows. Within the global energy and dissipation constraints, the gradients (*d*/*dy*+ or *d*^2^/*dy*+^2^) of the Reynolds stress components neatly fold onto respective curves, so that function prescriptions (dissipation structure functions) can serve as a template to expand to other Reynolds numbers. The Reynolds stresses are fairly well prescribed by the current scaling and dynamical formalism so that the origins of the turbulence structure can be understood and quantified from the entropy perspective.

## 1. Introduction

Turbulence, and its various aspects and applications, have been studied for over a hundred years. Yet, it remains an enigmatic topic in fluid mechanics and physics, in spite of numerous ideas, experiments and, numerical simulations [1]. We can think of three essential elements in turbulence: Dynamical closure (mostly modeling), structural scaling, and energy spectra. The third problem is sometimes referred to as the spectral closure, while its dynamical counterpart is an attempt to determine the Reynolds stress in terms of other root turbulence variables. Since many problems of practical significance exist, an encyclopedic volume of “turbulence models” evolved to carry out utilitarian and approximate simulations for turbulent flows. The ultimate in numerics is the direct numerical simulation (DNS), which produces an abundance of turbulence data [2,3]. These are very useful, but should be concurrent with fundamental attempts to theoretically solve the problem. Fife and co-workers, for example, published a number of papers on asymptotic layer analysis [4,5,6] and furnished some insights. The attached eddy hypothesis receives popular support, as reviewed in Smits et al. [7] and Marusic and Monty [8]. A lesser-known series of work has made some advances in this regard as well. By casting the momentum and energy conservation laws in a coordinate frame moving at the local mean velocities, decoupling of the mean and fluctuation variables resulted, leading to a closed set of transport equations [9,10,11,12]. The flux dynamics encapsulated by this alternative dynamical theory provide complete and succinct prescriptions of the turbulence structure, in canonical flow geometries. During this analysis, a spin-off idea of scaling the gradients has been inspired, leading to the self-similarity in the dissipation structure of the Reynolds stress tensor components, *u′*^2^, *v′*^2^, and *u′v′* [11].

In most of the analyses above, attention has been focused on the use of the momentum conservation (Navier–Stokes equations) and energy principles, but not as much on the entropy. For determination of the turbulence energy spectra, for example, the well-known Kolmogorov theory [13] starts with the energy content at a given scale, then uses dimensional arguments to construct a power-law type of energy transfer in the so-called ‘inertial range”. Inter-scale transport theories, such as the eddy-damped quasi-normal Markovian (EDQNM) theory, introduce eddy-to-eddy momentum transport models to complete the spectral closure [14,15]. There exists a set of works in the literature invoking the maximum-entropy principle to derive the turbulence energy distribution; however, they again rely on the scale-to-scale transport concept to constrain the spectral function. In recent monographs, lognormal-type spectra are derivable from the maximum-entropy principle, which agrees quite well over the entire range of turbulence scales and for different types of flows [16,17]. Furthermore, the turbulence structure in some basic geometries can be understood in terms of the energy and entropy (dissipation) concepts constrained by the boundary conditions of the flow [9,10,11,12]. In this work, we would like to explore the connection between entropy and turbulence in both its spectral and spatial distributions.

## 2. Turbulence Energy Spectra and the Maximum Entropy Principle

The maximum entropy principle states that the energy distribution tends toward an equilibrium state of maximum entropy while constrained by the boundary conditions. For turbulence, this translates to the observation that the energy spectra will assume a distribution with the maximum entropy under physical constraints, such as the zero energy at the boundaries and fixed total energy content. The kinetic energy approaches zero at the smallest dissipation scale (Kolmogorov scale), and also near the largest flow length scale (dimension of the object in the flow). The length scales in turbulence ranges from Kolmogorov (η) to the integral scales (*l*_e_), and the ratio is known from η = *l*_e_Re_λ_^3/2^ [6], where Re_λ_ is the Reynolds number based on the Taylor microscale (λ). Furthermore, conservation of energy is stipulated: The kinetic energy can cascade from one eddy scale to another and is dissipated by viscosity, but the total energy content remains constant. A distribution function that satisfies these criteria under the maximum-entropy principle is the lognormal function due to its asymmetric decay to zero at the boundary points. Uniform and exponential distributions have finite boundary values, while Gaussian is symmetric. Asymmetry in the energy spectra arises due to the descent toward zero energy at the low wavenumbers, while viscous dissipation reduces the kinetic energy at high wavenumbers. For a similar reason, the drop size distributions in spray flows take on a lognormal shape [18]. As noted above, the ratio of turbulence length scales specifies the width of the distribution through η = *l*_e_Re_λ_^3/2^, while the height of the distribution is set by the total integrated turbulence kinetic energy. 

Mathematically, the Lagrange multiplier method is applied to derive the energy distribution that maximizes Shannon’s entropy under the physical constraints [19]. For turbulence energy spectra, the principal constraint is the energy principle: The kinetic energy is dissipated by the viscosity effect progressively at large wavenumbers [20].

(1) $${u^{\prime }}^{2}+\nu k^{2}{u^{\prime }}^{2}\delta t=e_{o}=constant$$

*u*′(*k*) is the turbulent fluctuation velocity at a given wavenumber, *k*, while *ν* is the kinematic viscosity during some time interval, *δt*. Equation (1) states that the turbulence energy density (on a unit-volume basis) integrated over some time interval *δt* is conserved. The above constraint can be transposed into the energy distribution using the Lagrange multiplier method [19]. The first step is to write the objective function *F* so that

(2) $$F={u^{\prime }}^{2}+\nu k^{2}{u^{\prime }}^{2}\delta t-e_{o}$$

This is the standard stipulation of the constraints in the maximum-entropy formalism [19]. The most probable distribution function is found by maximizing log*F*, following the concept of Shannon’s entropy, *S* = *F*log*F* [19]. Using the Lagrange multiplier method, this distribution has an inverse exponential form [16,17].

(3) $$E\left(k\right)dV=C_{1}exp\left\{-C_{2}{u^{\prime }}^{2}-C_{3}k^{2}{u^{\prime }}^{2}\right\}dV$$

The Lagrange multipliers, *C*_1_, *C*_2_, and *C*_3_ are determined from other constraints. For example, *C*_1_ is determined by integrating *E*(*k*) to equal the total energy content in the distribution. *C*_2_ and *C*_3_ are determined from the limiting length scales and viscosity, respectively. Conversion of the volume to wavenumber space, *dV* = *d*(*k*^−3^), results in the following energy distribution:

(4) $$E\left(k\right)=\frac{C_{1}}{k^{4}}exp\left\{-C_{2}{u^{\prime }}^{2}-C_{3}k^{2}{u^{\prime }}^{2}\right\}$$

We still have the unknown kinematic scaling for *u*′(*k*) in Equation (4). Observational data are used to deduce the empirical form for *u*′(*k*)~(m-log(*k*)), where m is the logarithmic mean wavenumber [17]. In comparison, the Kolmogorov theory [6] results in *u*′~*k*^−1/3^ in the inertial subrange [20]. 

**Figure 1:**
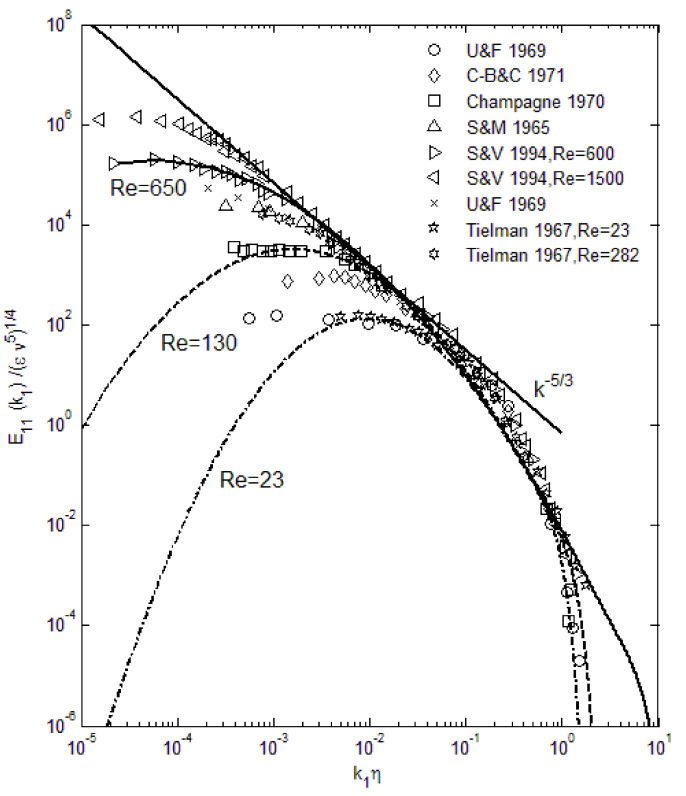
Comparison of the turbulence energy spectra using Equation (4) with experimental data. Various Reynolds numbers are included from Re_λ_ = 23 to 650. Kolmogorov scaling (*k*^−5/3^) is also plotted. Symbols are data [24,25,26,27,28,29].

The above spectral function (Equation (4)) exhibits all the attributes of the turbulence energy distribution as shown in Figure 1, and also produces excellent agreement when compared with data over the entire wavenumber range. In Figure 1, we can see that the viscous effect starts to be significant at high wavenumbers, where it causes a rapid decay toward zero energy. This is observable in some experimental data. Can this approach be applied in other types of turbulence? Considerations of the maximum-entropy formalism and early indications are positive in this regard. In atmospheric boundary layers, turbulence tends towards a two-dimensional geometry due to the large ratio of horizontal to vertical length scales, and researchers have been puzzled over the different exponents in the Kolmogorov type of scaling [21]. However, this is seen as the manifestation of the reduced range of length scales in two-dimensional turbulence [17]. The maximum-entropy method is applicable to energy spectra in any physical processes, such as photons, molecules, and turbulence eddies, so that with appropriate constraints, other types of turbulence such as magnetohydrodynamics may potentially be treated in a similar manner. As noted earlier, the use of the maximum-entropy principle in turbulence is not completely new, except that constraining by the momentum equation leads to complex mathematical derivations and mostly replicates the Kolmogorov type of scaling [22,23]. The main reason for this outcome is that the momentum conservation alone does not provide sufficient information concerning the energy transfer or its distributions. As noted above, the maximum-entropy principle is most effective when used in conjunction with the energy constraints. 

**Figure 2:**
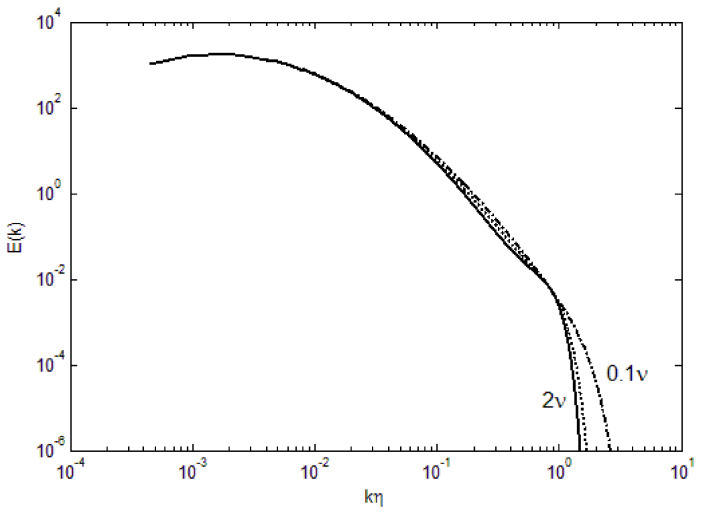
Effect of viscosity on the turbulence energy spectra.

We can further examine the effect of various parameters in the turbulence energy spectra. For small *ν* (kinematic viscosity), the deviation from the lognormal shape is minimal, as shown in Figure 2. With increasing viscosity, the bend toward zero energy becomes more abrupt at high wavenumbers. The cut-off wavenumber for this bend does not appear to change appreciably with the viscosity, although at 2*ν*, the effect of viscosity is transmitted to mid-range wavenumbers. Furthermore, with increasing viscosity, there is a flattening from the lognormal curve at the mid-wavenumbers, resembling the Kolmogorov’s *k*^−5/3^ scaling in the so-called inertial range. However, due to the spectral shape, any tangential scaling law can be fit; for example, *k*^4^ dependence has been suggested in the ascending part of the spectra [20], while *k*^−3^ has been observed for two-dimensional atmospheric turbulence [21]. Due to the compaction in the length scales, a steeper spectral shape is also found in the current derivation (Equation (4)).

## 3. Turbulence Structure in Channel Flows

**Figure 3:**
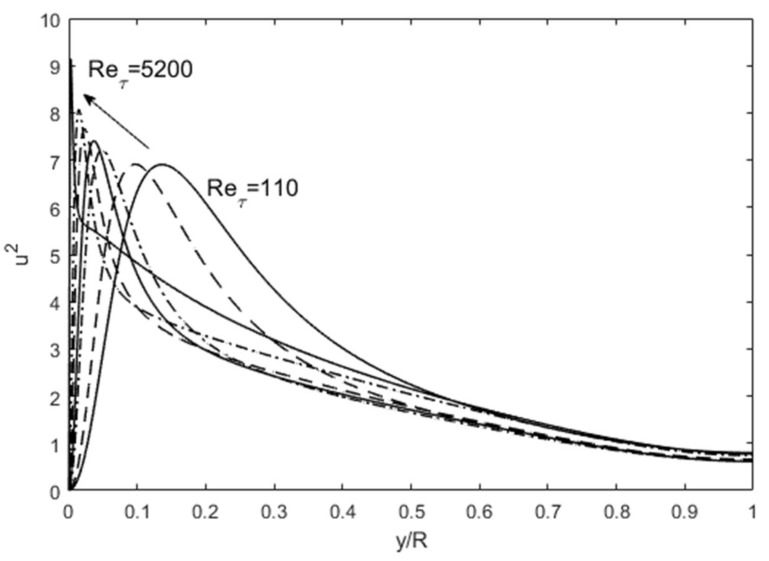
The progression of *u*′^2^ profiles with increasing Reynolds number. DNS data are from Iwamoto et al. [30] and Graham et al. [31].

**Figure 4:**
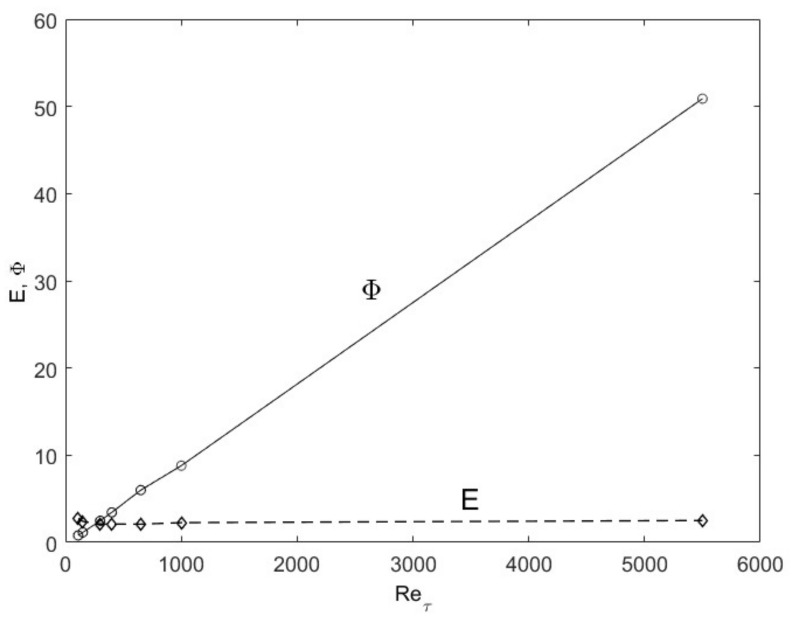
The total turbulence kinetic energy (E) and dissipation (Φ) as a function of Reynolds number for channel flows. DNS data from Iwamoto [30] and Graham et al. [31] are used.

The turbulence structure in channel flows can also be viewed from an entropy perspective. The spatial distribution of *u*′^2^ (turbulence kinetic energy) in wall-bounded (channel and boundary-layer over a flat plate) flows undergoes a progression with an increasing Reynolds number as shown in Figure 3, wherein the peak *u*′^2^ moves close to the wall with a sharp gradient followed by a gradual descent toward the centerline boundary condition. Scaling of these profiles has been attempted with various methods, including the attached-eddy hypothesis [8]. Globally, some predictable patterns emerge if we consider the total integrated turbulence kinetic energy (E from Equation (5)) and dissipation (Φ from Equation (6)). In Figure 4, the total integrated turbulence kinetic energy, E, normalized by the friction velocity (*u*′^+^ =*u*′/*u*_τ_ where *u*_τ_ is the friction velocity), is constant. On the other hand, the dissipation, Φ, exhibits a linear dependence on the Re_τ_. The entropic interpretation is that for fixed normalized energy content, E, dissipation increases because the relative influence of viscosity is reduced at higher Reynolds numbers. The u′^+2^ distribution distorts itself to achieve the maximum dissipation until the restraining force of viscosity imposes its upper limit, all the while obeying the physical constraints. The u′^+2^ profiles are physically and mathematically smooth and continuous functions, while constrained by the constant energy (E) and boundary conditions at the wall and the centerline (see Figure 3). The maximum distortion, which may be interpreted as turbulence entropy, is set by the viscous dissipation, Φ. Therefore, the origin of the turbulence structure can also be attributed to the Second Law. After this manifestation, other turbulence variables organize themselves according to the momentum (Equations (7) and (8) and energy (Equation (9)) conservation principles.

(5) $$E_{}=\int_{0}^{1}{u^{\prime }}^{+2}\left(y\right)d\left(\frac{y}{d}\right)$$

(6) $$\Phi =\int_{0}^{1}{\left(\frac{d{u^{\prime }}^{+}}{dy}\right)}^{2}d\left(\frac{y}{d}\right)$$

*u*′ momentum transport:

(7) $$\frac{d\left(u^{\prime }v^{\prime }\right)}{dy}=-C_{11}U\frac{d\left({u^{\prime }}^{2}\right)}{dy}+C_{12}U\frac{d{v^{\prime }}^{2}}{dy}+C_{13}\frac{d^{2}u^{\prime }}{dy^{2}}$$

*v*′ momentum transport:

(8) $$\frac{d\left({v^{\prime }}^{2}\right)}{dy}=-C_{21}U\frac{d\left(u^{\prime }v^{\prime }\right)}{dy}+C_{22}U\frac{d{v^{\prime }}^{2}}{dy}+C_{23}\frac{d^{2}v_{rms}^{}}{dy^{2}}$$

*u*′^2^ transport:

(9) $$\frac{d\left({v^{\prime }}^{3}\right)}{dy}=-C_{31}\frac{1}{U}\frac{d\left(u^{\prime }v^{\prime }\cdot u^{\prime }\right)}{dy}+C_{32}\frac{1}{U}\frac{d\left(v^{\prime }\cdot u^{\prime }v^{\prime }\right)}{dy}+C_{33}\frac{1}{U}{\left(\frac{du^{\prime }}{dy}\right)}^{2}$$

In Equations (7)–(9), the fluctuating terms, *u*′*v*′, *u*′^2^, *v*′^2^, etc., are Reynolds-averaged turbulence velocity fluctuations, and *C*_ij_ is a displacement constant with the unit of inverse velocity (s/m). *y*+ is the so-called “inner coordinate” for turbulent boundary layers. The concepts and hypotheses contained in Equations (3)–(5), and their efficacy in prescribing the Reynolds stress tensor, are described in Lee [10,11,12]. We can see that Equations (7) and (8) are momentum-conserving, while Equation (9) is an expression of energy balance. The transport equations involve gradients up to the second, as flux or viscous terms. 

**Figure 5:**
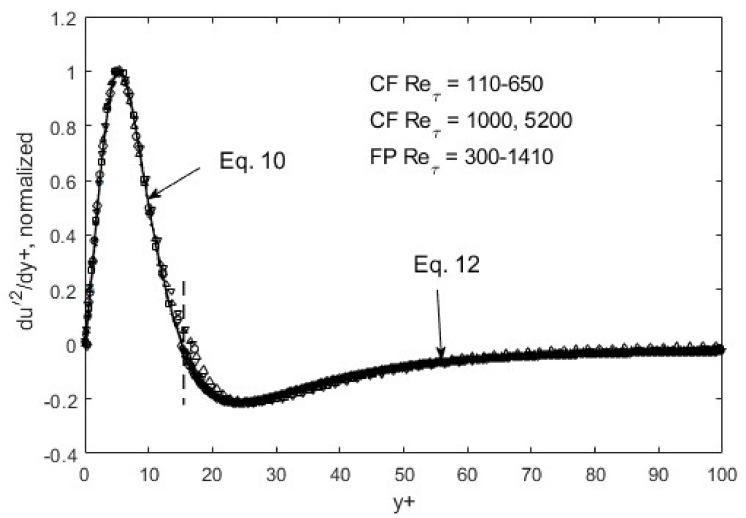
du′^2^/dy+ normalized and ratioed (to preserve the overall function shape) by respective peak/nadir magnitudes left and right of zero-crossing (*y*+~15). CF = channel flow, DNS data from Iwamoto et al. [30] and Graham et al. [31]; FP = boundary-layer flow over a flat plate, DNS data from Spalart [32].

**Figure 6:**
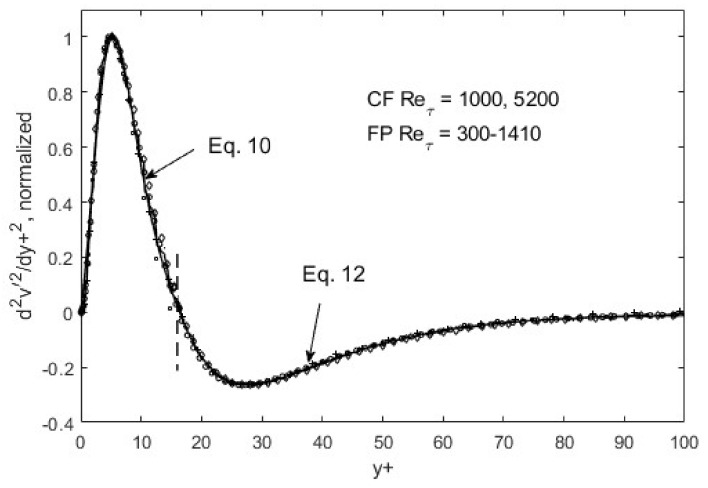
*d*^2^*v*′^2^/*dy*+^2^ normalized and ratioed (to preserve the overall function shape) by respective peak/nadir heights left and right of zero-crossing (*y*+~10). CF = channel flow, DNS data from [31]; FP = boundary-layer flow over a flat plate, DNS data from Spalart [32].

**Figure 7:**
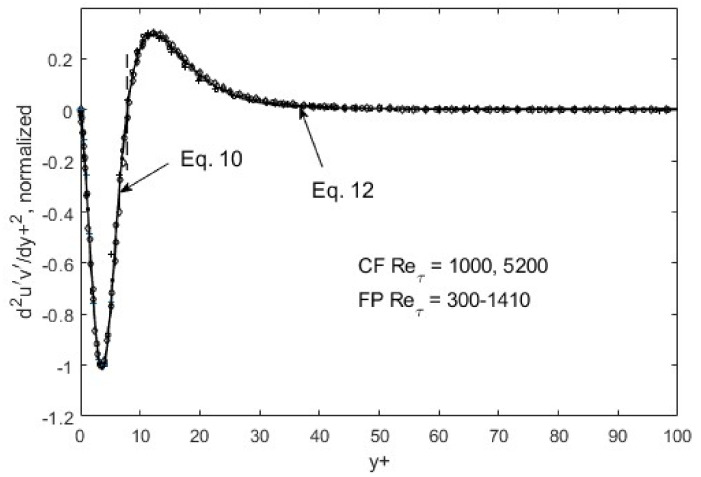
*d*^2^*u*′*v*′/*dy*+^2^ normalized and ratioed (to preserve the overall function shape) by respective peak/nadir heights left and right of zero-crossing (*y*+~10). CF = channel flow, DNS data from Graham et al. [31]; FP = boundary-layer flow over a flat plate, DNS data from Spalart [32].

Within the above global constraints, the resulting turbulence structure exhibits fully scalable patterns, as shown in Figure 5, Figure 6 and Figure 7. That is, self-similarity exists at the first gradient level for the *u*′^2^ distribution in space. If we plot *du*′^2^/*dy*+ and normalize the maxima and minima as in Figure 5, all the data collapse onto a single curve for both channel (CF) and flat-plate boundary layer flows (FP). Similar scaling for the other Reynolds stress components can be found for *v*′^2^ and *u*′*v*, except at the second-gradient level (Figure 6 and Figure 7).

The self-similarity mentioned above evidently means that a single representation suffices for each of the root turbulence variables, again at the appropriate gradient levels [11]. Then, along with scaling factors, e.g., maximum and minimum *du*′^2^/*dy*+ as a function of the Reynolds number, *u*′^2^, *v*′^2^, and *u*′*v*′ can be reconstructed. The peak and nadir magnitudes of the gradients of *u*′^2^, *v*′^2^, and *u*′*v*′ vary asymmetrically as a function of the Reynolds number [11,12], yet accounting for this variation allows for a single profile to represent the gradient structure across both channel (CF) and flat-plate (FP) flows. The observed peak location (*y*+~15) and other features of *u*′^2^ spatial distributions appear to be manifestations of this “gradient structure”. The scalability uniquely captures the essential features of wall-bounded turbulent flows across the entire width of the boundary layer. Furthermore, similar scaling in *v*′^2^ and *u*′*v*′ are found, except at the second-gradient level [11,12]. We observed in Figure 1 and Figure 2 that although *u*′^2^ profiles are difficult to trace using standard functions, particularly at high Reynolds numbers, their gradients are quite well-behaved, even looking familiar. On the near-wall side, prior to the zero-crossing point (*y*+~15), the *du*′^2^/*dy*+ profile is traced by a “modified” Gaussian function (Equation (10)), while on the aft side, an exponentially decaying sinusoidal function (Equation (12)) suffices to approximate the DNS data. Parametric modifications are made in these functions to capture the structural curves of the gradients, and the parameters are listed and tabulated in Table 1.

Modified Gaussian function (prior to zero-crossing):

(10) $$f\left(x\right)=y_{o}+\frac{A}{t_{0}}e^{\left(\frac{1}{2}{\left(\frac{w}{t_{0}}\right)}^{2}-\frac{x-x_{c}}{t_{0}}\right)}\frac{1}{2}\left(erf\left(\frac{z}{\sqrt{2}}\right)+1\right)$$

where *erf* is the error function and

(11) $$z=\frac{x-x_{c}}{w}-\frac{w}{t_{0}}$$

Exponentially decaying sinusoid (aft of zero-crossing):

(12) $$g\left(x\right)=y_{o}+Ae^{-\frac{x}{t_{0}}}\sin\left(\pi \frac{x-x_{c}}{w}\right)$$

The functions, Equations (10) and (12), are piece-wise continuous and smooth, and represent inverse solutions to the transport equations (Equations (7)–(9)). Some may refer to it as curve-fitting, which may be appropriate if separate tracing was needed for different Reynolds numbers; however, since only one set of function representations suffices, as shown in Figure 5, Figure 6 and Figure 7, Equations (10) and (12) constitute an inverse solution to the transport equations, Equations (7)–(9). One of the solution functions is the second gradient of the Reynolds shear stress, so that upon integrations, it produces *u*′*v*′ = *f*(*y*+) that can be entered into RANS to generate the mean velocity profile. As is well known, the RANS (Reynolds-averaged Navier–Stokes) equation in and of itself is not solvable due to the unknown Reynolds shear stress term. The transport equations (Equations (7)–(9)) and/or the gradient structure functions (Equations (10)–(12)) furnish the supplementary dynamics leading to the Reynolds shear stress, enabling RANS solutions. 

## 4. Concluding Remarks

The links between entropy and the turbulence structure are examined for spectral and spatial distributions of turbulence energy. The First and Second laws of thermodynamics govern all the processes in the universe, and turbulence is no exception. If we depart from the conventional turbulence theories and view this phenomenon from the entropy perspective, clear and logical explanations of the observed structure can be discovered. For example, in place of the partial and arguable Kolmogorov *k*^−5/3^-law, log-normal type distributions derivable from the maximum entropy principle prescribe the complete energy spectra. Spatial structure can also be viewed from the entropy or dissipation concept, wherein the viscous and wall-boundary effects enforce constraints on the possible energy distributions and the remaining elements are dictated by momentum and energy transport processes. The fully scalable turbulence profiles can be summarized in dissipation structure functions for each of the Reynolds stress components.

## Figures and Tables

**Table 1:** Coefficients used in the functions (Equations (10) and (12)) for *du*′^2^/*dy*+ (Figure 5), *d*^2^*v*′^2^/*dy*+^2^ (Figure 6), and *d*^2^*u*′*v*′/*dy*+^2^ (Figure 7).

		*y_o_* (Offset)	*A* (Amplitude)	*x_c_* (Phase Shift)	*w* (Period)	*t*_0_ (Decay Constant)
$\frac{du\prime^{2}}{dy^{+}}$	Equation (1)	−0.4585	19.086	2.270	2.744	6.933
	Equation (2)	−0.0258	5606.2	−67343	67358	9.742
$\frac{d^{2}v\prime^{2}}{dy^{+2}}$	Equation (1)	−0.3633	17.453	2.384	2.556	7.041
	Equation (2)	−0.0095	2432.8	−29463	29480	11.32
$\frac{d^{2}u\prime v\prime }{dy^{+2}}$	Equation (1)	0.26134	−7.065	2.564	1.867	1.395
	Equation (2)	0.00437	3.153 × 10^7^	7.885	8.686 × 10^7^	4.307

## Data Availability

Not applicable.
